# Surgery

**Published:** 1898-06

**Authors:** Charles A. Morton, James Swain


### SURGERY.

As sarcoma of the kidney is frequently found in young children, a recent paper[3] by Dr. George Walker, of the Johns Hopkins Hospital, is of great interest and importance. It is based on the records of 145 cases of **sarcoma of the kidney in children.**

The **pathology** of these tumours is of considerable interest. They are mixed tumours—growths composed of more than one kind of tissue—and hence are regarded by most pathologists as embryonic in origin. In fact it was a study of one of these tumours which led Cohnheim to form his embryonic theory of the origin of all tumours. In these growths we find striated muscle tissue, cartilage, and adenomatous tissue. The striped muscle fibres are found in one-fourth of all cases microscopically examined. The fibres are more like embryonic striated muscles

[3] *Ann. Surg.*, 1897, xxvi. 529.

than the fully-developed tissue. In one case they have even been found in a secondary growth. The adenomatous tissue resembles more the Wolffian body than mature renal tissue. Its presence has led some of the older pathologists to classify these growths as carcinoma.

Secondary growths have occasionally been found in the retroperitoneal and mesenteric glands, but as a rule the lungs and liver are affected. It is an interesting fact that secondary growths are very rare in the ureter or bladder. In some of our English text-books, the other kidney is said to be very often the seat of sarcoma also (in one text-book it is stated that this is so as often as in half the cases); but this statement is not substantiated by the study of the 145 recorded cases, although a few cases of bilateral renal sarcoma are referred to.

The tumours in many cases attain an enormous size, and in several have been more than half the weight of the child after removal of the growth. They are frequently covered with large veins, either emerging from the growth or forming a plexus in the overlying peritoneum. In some cases the soft tumour tissue bursts through the capsule and leads to lobulation of the growth. The kidney is not wholly destroyed in all the cases; in a few it has projected in part or as a whole from the growth, but in no less than twenty was a portion of the kidney found under the capsule of the growth, and in two cases the whole kidney was found beneath the capsule. The appearance of the tumour on section varied greatly. In many cases various forms of degeneration were present; and in a few cysts were discovered, many quite small and associated with the adenomatous growth; but in one case a cyst of very large size was present, and in several there were degeneration cysts of considerable size, or large areas of the growth consisted of extravasated blood and broken-down tumour tissue.

The manner in which these tumours displace the contents of the abdominal cavity is important. A large growth of the right kidney has been known to raise the diaphragm up to the fourth rib. The colon is of course raised by the tumour, but if the growth attains a great size it is pushed inwards as well, and this is particularly apt to occur on the right side, the colon thus forming a line from the cæcum to the splenic flexure. When the colon or other portion of bowel lies on the top of a very large growth it may be so pressed against the abdominal wall that it contains no gas, and hence does not yield a tympanitic note, but may be felt as a thick band rolling under the fingers over the tumour, as was the case in a patient under my own care.

From the point of view of the operating surgeon, the presence or absence of important adhesions is a matter of much moment. Fortunately the proportion of cases with adhesions is not very large, and when they occur they are usually simply peritoneal; any of the neighbouring organs may be adherent

(most frequently the stomach), but it is interesting to note that in only five cases is the vena cava said to have been adherent. In two cases the growth had eroded the vertebræ and caused symptoms of pressure on the cord. A turbid brown fluid in varying amount was usually found in the peritoneum.

The well-recognised symptoms of malignant renal growth were present in these cases of sarcoma in children, and it is important to observe that in the majority the first symptom noticed was the presence of the abdominal swelling; but in a fair number hæmaturia was the first symptom. Vomiting has been present in many cases.

The differential diagnosis of these tumours is carefully considered by Dr. Walker, but the only point that I need refer to is the great difficulty of distinguishing them from malignant growths in the retro-peritoneal glands; but this condition is very rare. When the tumour has been explored by an aspirating needle, only a few drops of bloody serum have been obtained in the recorded cases collected by Dr. Walker; but in a case lately under my own care, after exploration of the growth by an abdominal incision, the needle of an exploring syringe was introduced into it from the loin, and a syringeful of dark-brown clear fluid was obtained. Although the colour of the fluid was unlike that of hydronephrosis, the fact that a syringeful was withdrawn suggested this condition; but a lumbar exploration showed that the mass was sarcoma, the fluid being probably the result of previous hemorrhage into the growth.

These tumours of the kidney are most frequently met with in young children, between one and five years of age.

The result of **removal of the growth** has been very discouraging—so much so, that some surgeons who have studied the mortality statistics have formed the opinion that they had better be left alone. There are only four cases on record in which the child has lived for three years after removal of the growth, *i.e.*, four cases of "cure" according to the three years rule. Two of these are cases operated on by Abbe, of New York, and in one of these the growth was so large as to nearly fill the abdomen. It weighed 7½ lbs., *i.e.* half the weight of the child, after its removal. Microscopic examination showed it to be a sarcoma containing striated muscle fibres. The child was well 3½ years later. The other two cases of "cure" were operated on by two German surgeons, Israel and Schmidt.

The **mortality** statistics collected by various surgeons during the last ten years have varied considerably, but they have all been high—some so high as to place the operation, in the opinion of the surgeons who have brought them forward, outside the range of justifiable surgery. There is one hopeful fact, and that is, that the mortality has diminished of late years. Dr. Walker gives us the mortality as 38.25 per cent. Most of the deaths are from shock. The removal of the growth is said to prolong life from 8.08 (the average duration without operation)

to 16.77 months. He says: "Although the cures are very few, still in consideration of the invariably fatal termination of this malady, without interference, I unhesitatingly advise operation, for it offers the only hope; and at worst it means only an accelerated death." With this I cannot altogether agree. It means the chance of immediate death in about one case in every three operated on; a prolongation of life by some months in those patients who survive; and a so-called cure in only four cases out of 145 recorded. I think it is a matter for the parents to decide whether the child shall incur the risk for the chances offered; and I do not think that nephrectomy could be recommended, except in an early stage when the tumour was quite small.

Dr. W. B. Coley, of New York, has recorded[1] 250 cases of **radical cure of hernia in children.** Bassini's method was adopted. There was only one fatal case—a child died from pneumonia, due, Coley thinks, to the administration of ether for anæsthesia. He considers that it is a good rule never to advise the operation in children until a truss has been tried for one or two years without benefit; and his 250 cases of operation were selected from 7000 cases of ruptured children under 14 years of age. He raises the important question as to how many persons who have hernia in infancy and childhood remain uncured in adult life? He has collected records of 400 such cases during the last four years in his practice as surgeon to the Hospital for Rupture in New York. He claims that the mortality of operation for radical cure in childhood is less than in adult life, and is as low as half to one per cent. in a collection of no less than 832 cases. He considers that cases of hernia in children are more easily cured by operation than in adults, just as they are by wearing a truss. Many of the cases operated on by Coley have now remained free from recurrence for several years; indeed, out of 280 cases of Bassini's operation which he has been able to trace, in only three has relapse occurred. In fourteen cases the patients were examined four to five years after operation, and no recurrence was found. These cases include adults as well as children.

At a meeting of the New York Surgical Society on April 14th, 1897,[2] Dr. Coley reported a case of double femoral hernia in a male child aged two years. On the right side the cæcum, uncovered by peritoneum on its posterior aspect, was found in the sac. Femoral hernia in children is very rare.

In Bassini's operation for the radical cure of hernia the cord is transplanted on to the surface of the internal oblique, and is still covered by the external oblique aponeurosis; in Halsted's operation the cord is placed on the outer surface of the external oblique. Dr. G. Ryerson Fowler, of Brooklyn, has devised a

[1] *Ann. Surg.*, 1897, xxv. 270. [2] *Ibid.*, xxvi. 246.

new operation for radical cure of hernia, in which he transplants the cord within the peritoneal cavity, by slitting up the posterior (peritoneal) layer of the inguinal canal.[1] He makes the cord emerge from the peritoneum in the position of the external ring, and he attempts to strengthen the wall at this point by trying to drag the rectus muscle over its point of exit. I fail to see that hernia is less likely to occur here than in the position of the internal ring, and do not believe that the rectus can be dragged over the new point of exit of the cord from the peritoneum in the way described. It seems to me that hernia is much less likely to occur if the exit of the cord is left in the usual position, with the external oblique carefully sutured over it. Dr. Fowler has not operated on enough cases, or for a long enough period, to show his results.

We are all familiar with acute infective inflammation of the long bones and their periosteum in children, but probably few of us have studied the disease which has lately been described by Makins and Abbott[2] and by Chipault[3] as **acute primary osteomyelitis of the vertebræ.** Records of twenty-eight cases of this rare but important disease have been collected. It is essentially a disease of childhood—the larger number of the cases occurred between the ages of ten and fifteen years. The general symptoms are those of an acute infective inflammation of bone, and the local signs are inflammatory induration and tenderness about some definite area of the spine, with the evidence of the formation of abscesses at a later date. When this abscess is opened the bare portions of the spine are discovered. At the commencement of the disease there may be only a tendency to assume the supine posture, with tenderness and rigidity of the affected portion of spine; then œdema may be observed, followed by widespread inflammatory induration, and finally by abscess. If the body of the vertebræ, rather than the neural arch, is affected, the local swelling in the back may not be present, and the pain on movement, rigidity of the spine, and general symptoms of acute inflammatory disease may be all we have to depend upon for diagnosis. The pus may occupy the positions in which tuberculous abscesses from Pott's disease are found—double psoas abscess, for instance, may be produced. If the disease attacks the neural arch, abscess will form in the back; if the body, the abscess will form behind the pharynx, in the mediastinum, or in the psoas muscles, according to the level of the disease. In several cases spinal meningitis has been set up, and Makins and Abbott suggest that acute osteomyelitis has been overlooked as the cause of meningitis in a considerable number of instances of so-called acute spinal meningitis or myelitis, and they state that "in rare cases the first notable

[1] *Ann. Surg.*, 1897, xxvi. 603. [2] *Ibid.*, 1896, xxiii. 510.
[3] *Gaz. d. Hôp.*, 1896, lxix. 1393; abstract in *Med. Chron.*, 1896-97, N.S. vi. 272.

symptoms have been those due to implication of the cord or meninges."

As might be expected, excurvation of the spine was only observed in two cases, and in these disappeared. The lumbar vertebræ are more frequently the seat of this disease than those in the cervical or dorsal regions; and when they are affected on their anterior aspect, pain in, and distention of, the abdomen are often marked symptoms, and may simulate peritonitis. This is probably due to early implication of the psoas muscles, before they become converted into palpable abscess sacs; but it is thought possible that irritation of the nerve-roots and sympathetic ganglia of the abdomen may be to some extent the cause of these symptoms. Vertebral osteomyelitis seems to have been uniformly fatal without surgical treatment, but of sixteen cases so treated eight have recovered. Abscesses must of course be opened early, but they are difficult of detection, even in the back, and much indurated tissue may have to be cut through before pus is reached. Sequestra of the normal arches should be removed. If symptoms of spinal meningitis are present, the spinal canal must be opened up by the removal of diseased or even healthy bone.

CHARLES A. MORTON.

Kraske[1] has published his experiences of the operation which he proposed in 1885 for **cancer of the rectum.** Of 110 cases 80 were subjected to operation. The tumour was more frequently found in the upper portion of the rectum, necessitating the opening of the peritoneum during operation in two-thirds of the cases. The average duration of life of the round-cell form—which is nearly always the type found—must be placed between four and five years. The fact that the subject is not of advanced years is no proof against the existence of cancer, for he has observed it in a man twenty-three years old, and I have had under my care a patient who was only twenty-one years of age. It is of more relative importance to ascertain the degree of mobility of the tumour than to define its upper limit; for opening of the peritoneum is no longer a contra-indication to operation, and, moreover, it is extremely rare to encounter a cancer of the rectum longer than ten to twelve centimetres. The performance of colotomy is only indicated for the relief of complete or impending obstruction, for such cases only drag out a miserable existence with an artificial anus. The high situation of a cancer of the rectum is no contra-indication to operation, for a tumour in the uppermost portion can be removed without difficulty, and the extirpation of a growth situated in the intra-peritoneal portion of the bowel is easier than of one situated lower down, provided that it is freely movable. The thorough evacuation of the contents of the intestines by

[1] *Samml. klin. Vortr.*, Nos 183, 184; editorial in *Ann. Surg.*, 1897, xxvi. 371.

cathartics and enemata previous to operation—often necessitating three or four weeks of treatment—is very important as a preparatory measure, and the author thinks that the failure to properly carry out this preparation is responsible for some of his fatal cases. In operating, the patient is placed on his right side, and the incision now adopted begins at the level of the middle of the sacrum, two or three fingers' breadth to the left of the median line, and runs slightly concave to the left, to the coccyx, running from that point in the median line, and ends a finger's breadth above the anus. The soft parts and the insertion of the sacro-iliac ligaments are divided, and after cutting through the loose fat in the ischio-rectal fossa, the levator ani and coccygeus are divided close to the sacrum and coccyx. Resection of a portion of the sacrum is now necessary, and may be performed with chisel, bone-forceps or chain-saw, after separation of the soft parts in a line concave inward and downward, running from the third left sacral foramen to and around the fourth foramen to the lower angle of the sacrum. Transverse resection of the sacrum, as suggested by Bardenheuer, is only used for the more extensive cases. Rose makes a transverse section of the sacrum at the level of the second foramen, but this Kraske rejects. The attempted modifications in the direction of preservation of the bony structures, by temporary or osteoplastic resection, are founded on better judgment; but the author regards them as more serious and unnecessary, for he has never seen any disturbance of function arise from the separation of the sphincteric insertion to the sacrum, or that the floor of the pelvis has lost its support with a resulting prolapse of pelvic organs. The osteoplastic method will however probably be improved, and the best results may eventually be expected from a combination of this and the original method. The resection of the cancer should begin with division of the bowel below the tumour by opening it transversely, and sutures are placed on the upper cut surface for traction purposes. The patient is then brought into the lithotomy position and the dissection proceeded with. Sometimes the peritoneum can be peeled off the bowel; but if necessary it must be opened, two fingers introduced, the gut pulled down and divided, and the sacral glands removed. Suture of the peritoneum is not to be thought of unless the opening is a very small one. After trying various ways of suturing the cut ends of the bowel, the author has returned to his early method of immediate circular suture, as a successful result is perfectly possible, if a sufficiently careful preliminary treatment has been carried out, and if the bowels are kept constipated for eight or ten days after operation. Packing of the wound with iodoform gauze should be adopted to prevent infection, and need not be removed till it shows a tendency to become loose, about the end of the first week. It is impossible to estimate the direct danger of the radical operation for cancer of the rectum. Dividing Kraske's cases into two periods,—the first five years when he was evolving the present improvements, and the last seven, we have: 1. Twenty-nine cases, ten deaths, mortality, 34.5 per cent. 2. Fifty-one cases, five deaths, mortality, 9.8 per cent. The average time required for complete closure of the wound was four to six weeks. Excluding two cases of sarcoma, and three of squamous-cell cancer of the anus, observations of fifty-three cases are at hand. Twenty-two cases died of recurrence, with or without metastases, in from six months to twelve and three-quarter years after operation. Sixteen patients died from intercurrent diseases, without recurrence or metastases, from one and a quarter to five years after operation. Fifteen patients are alive and free from recurrence in from three-quarters of a year to eight and a half years after operation.

JAMES SWAIN.

